# Disruption of early visual processing in amyloid-positive healthy individuals and mild cognitive impairment

**DOI:** 10.1186/s13195-023-01189-7

**Published:** 2023-02-28

**Authors:** Daniel C. Javitt, Antigona Martinez, Pejman Sehatpour, Anna Beloborodova, Christian Habeck, Yunglin Gazes, Dalton Bermudez, Qolamreza R. Razlighi, D. P. Devanand, Yaakov Stern

**Affiliations:** 1 Division of Experimental Therapeutics, Department of Psychiatry, Columbia University Irving Medical Center, 1051 Riverside Drive, Unit 21, New York, NY 10032 USA; 2 Division of Schizophrenia Research, Nathan Kline Institute for Psychiatric Research, Orangeburg, NY 10962 USA; 3 Cognitive Neuroscience Division, Department of Neurology, Columbia University Vagelos College of Physicians and Surgeons, New York, NY 10032 USA; 4 Quantitative Neuroimaging Laboratory, Department of Radiology, Weill Cornell Medicine, Brain Health Image Institute, New York, NY 10065 USA; 5 Area Brain Aging and Mental Health, Columbia University Irving Medical Center/New York State Psychiatric Institute, New York, NY 10032 USA

**Keywords:** Visual processing, Event-related potentials, Oscillations, Amyloid, Alzheimer’s disease

## Abstract

**Background:**

Amyloid deposition is a primary predictor of Alzheimer’s disease (AD) and related neurodegenerative disorders. Retinal changes involving the structure and function of the ganglion cell layer are increasingly documented in both established and prodromal AD. Visual event-related potentials (vERP) are sensitive to dysfunction in the magno- and parvocellular visual systems, which originate within the retinal ganglion cell layer. The present study evaluates vERP as a function of amyloid deposition in aging, and in mild cognitive impairment (MCI).

**Methods:**

vERP to stimulus-onset, motion-onset, and alpha-frequency steady-state (ssVEP) stimuli were obtained from 16 amyloid-positive and 41 amyloid-negative healthy elders and 15 MCI individuals and analyzed using time–frequency approaches. Social cognition was assessed in a subset of individuals using The Awareness of Social Inference Test (TASIT).

**Results:**

Neurocognitively intact but amyloid-positive participants and MCI individuals showed significant deficits in stimulus-onset (theta) and motion-onset (delta) vERP generation relative to amyloid-negative participants (all *p* < .01). Across healthy elders, a composite index of these measures correlated highly (*r* =  − .52, *p* < .001) with amyloid standardized uptake value ratios (SUVR) and TASIT performance. A composite index composed of vERP measures significant differentiated amyloid-positive and amyloid-negative groups with an overall classification accuracy of > 70%.

**Discussion:**

vERP may assist in the early detection of amyloid deposition among older individuals without observable neurocognitive impairments and in linking previously documented retinal deficits in both prodromal AD and MCI to behavioral impairments in social cognition.

## Background

Alzheimer’s disease (AD) is a devastating illness associated with amyloid and tau deposition and neurodegeneration. When present, amyloid is a strong contributor to neurodegenerative change [1]. Deposition of amyloid plaques, moreover, may precede symptom onset by several decades, providing a window during which preventative treatments might be initiated [2, 3]. In addition, the degree to which amyloid deposition affects brain function even in advance of the development of clinical symptoms remains an area of active investigation (e.g., [4]).

Definitive tests for amyloid deposition include ^18^F-florbetapir PET imaging or CSF Aβ amyloid determination [5, 6]. Nevertheless, there has been increasing attention over recent years on retinal pathology that may precede overt illness development and may therefore serve as a useful screening tool during the early stages of the illness. The interest in retinal imaging derived initially from the observation of Aβ deposition in the postmortem retina of individuals with established AD, followed by findings of retinal thinning in AD individuals, particularly involving the ganglion cell layers (GCL) and the optic nerve (rev. in [7–10]). GCL degeneration as detected by optical coherence tomography imaging has also been observed in asymptomatic first-degree relatives of AD individuals [11]. Retinal structural abnormalities also correlate with reduced amplitude of both the electroretinogram (ERG) [12, 13] and the cortically generated visual event-related potential (vERP; also termed visual-evoked potential (VEP)) [13] to pattern stimuli in individuals with established AD.

Recently, the ERG was also studied in cognitively intact older individuals who had elevated CSF amyloid (Aβ +) levels (preclinical AD patients) vs. those without significant Aβ findings (Aβ −), using a multi-focal ERG approach that may preferentially detect M-cell dysfunction. A model including the ERG measures strongly differentiated the groups, with sensitivity and specificity of 87 and 82% and area under the receiver operating characteristic (ROC) curve (AUC) = 0.84 [14]. In the sample, retinal thickness was normal, suggesting that functional changes may predate structural changes. A second recent study found reductions in both GCL thickness and ERG response and AUC = 0.9 for the separation of the Aβ + vs. Aβ − groups using the combined structural and functional metric [15]. To date, no comparable study has been conducted with cortical vERP, although studies have been performed using alternative stimuli such as changes in whole-field luminance [16].

In the cortical vERP approach, visual stimuli are presented as with ERG, but responses are recorded over the occipital scalp to detect activity generated in the primary and secondary visual cortex. Cortical vERPs are driven by the projection of RGC to the cortex via the magno-, parvo-, and konio-cellular selective layers of the lateral geniculate nucleus. Magnocellular projections show preferential sensitivity to low spatial frequency and low-contrast stimuli, whereas parvocellular responses show preferential sensitivity to high-contrast and high-spatial frequency stimuli [17]. In addition, the response pattern differs to low- vs. high-spatial frequency stimuli, such that responses to low-spatial frequency stimuli are dominated by an initial P1 response [18] that likely originates from the extrastriate cortex [19], whereas high-spatial frequency stimuli show an initial C1 response that localizes primarily to the primary visual cortex [18, 19].

As compared with ERG responses, cortical vERPs tend to be larger and may provide greater information regarding visual subprocesses, such as responses to static vs. motion stimuli [17]. Furthermore, cortical vERPs are more proximate to the behavioral consequences of early visual system dysfunction. For example, deficits in the generation of the initial visual P1 response have been linked to impairments in perceptual closure [20], visual working memory [21], social cognition [22], and reading [23] in schizophrenia, as well as cognitive impairments in disorders such as multiple sclerosis (MS) [24].

Here, we implemented a recently developed efficient vERP approach that has successfully differentiate schizophrenia and autism spectrum disorder and control individuals and correlates with face emotion recognition accuracy [25]. A key element of this approach is that motion onset is delayed relative to stimulus onset, which allows the two processes to dissociate, and a long total interstimulus interval is used (5 s) to prevent habituation of the motion-evoked response [26]. We evaluated vERP in 57 cognitively normal older individuals who were taking part in a prospective longitudinal study of aging that included PET amyloid imaging, along with 15 individuals diagnosed with mild cognitive impairment (MCI) drawn from ongoing clinical studies.

We used a time–frequency (TF) analysis approach in which vERPs were resolved into underlying frequency ranges [27]. In this paradigm, stimulus-onset responses have predominant power in the theta (4–7 Hz) frequency range, whereas motion-onset responses have power primarily in delta (1–4 Hz) [25], permitting further dissociation of the two response types. We also obtained steady-state visual-evoked potential (ssVEP) responses at alpha (10 Hz) frequency, which may serve as an index of function within the pulvinar nucleus of the thalamus [28]. Last, in a subset of individuals, we collected data on The Awareness of Social Inference Task (TASIT) [29], which serves as a well-validated test of social cognitive function [30, 31].

We hypothesized that Aβ + but cognitively normal healthy individuals (i.e., pre-symptomatic AD) would nevertheless differ from Aβ − individuals on cortical vERP measures, especially involving motion sensitivity, and would provide between-group separation values similar to those obtained in recent ERG studies. In schizophrenia, we have previously shown that deficits in early visual processing correlate with impairments in specific cognitive processes such as visual learning, attention-vigilance, speed-of-processing [28], perceptual closure [20, 32], passage reading [23, 33] and face emotion recognition [25, 34], and social cognition [35, 36], but not with other domains such as declarative memory or reasoning/problem-solving. In healthy aging, age-related reductions in social cognitive ability as measured by tests such as The Awareness of Social Inference Test (TASIT) are also unrelated to general cognitive ability or cognitive reserve [37–40]. Deficits in social cognitive processes have also been linked with loneliness, an inability to maintain a healthy relationship, and health problems, which are important concerns in aging [37, 38]. Here, we collected TASIT data on a subset of individuals participating in this study, based on the results of a parallel study in schizophrenia linking impaired social cognition function to impaired visual sensory processing [35, 36].

## Methods

### Participants

A total of 57 participants aged 55–80 were recruited from an ongoing study of cognitive aging, which assesses longitudinal function over time and includes measures of overall cognition and Aβ deposition. The study was reviewed by the Columbia University Institutional Review Board (protocol #7104). Consent was obtained according to the Declaration of Helsinki.

Participants were recruited from the National Institute of Aging grant R01 AG026158 entitled “Imaging of Cogniton, Learning and Memory in Aging.” In the referring study, participants were recruited using a market-mailing approach. Participants who responded to the mailing were telephone screened to ensure they met the basic inclusion criteria (right-handed, English speaking, no psychiatric or neurological disorders, normal or corrected-to-normal vision). All participants found eligible via the initial telephone screen were further screened in person with structured medical, neurological, psychiatric, and neuropsychological evaluations to ensure that they had no neurological or psychiatric disease or cognitive impairment.

The screening procedure included a detailed interview that excluded individuals with a self-reported history of major or unstable medical illness, significant neurological history (e.g., epilepsy, brain tumor, stroke), history of head trauma with loss of consciousness for greater than 5 min, or history of an axis I psychiatric disorder (APA, 1994). Individuals taking psychotropic medications were also excluded. Global cognitive functioning was assessed using the Mattis Dementia Rating Scale, on which a score of at least 135 was required for retention in the study [41].

In addition, a neuropsychological battery including the Selective Reminding Task (SRT) [42] established that participants were cognitively normal and did not meet the criteria for mild cognitive impairment (MCI). As part of the parent study, all participants were offered Aβ PET. During the period of the current study, all participants who had recently completed an Aβ PET scan were referred for the EEG studies.

In parallel, a group of 15 patients were recruited from the National Institute of Aging grant AG041795 entitled “Olfactory deficits and donepezil treatment in cognitively impaired elderly,” but were not on donepezil at the time of testing. Subjects were of similar age to the cognitively intact participants recruited from AG026158 but met the National Institute on Aging guidelines criteria for amnestic MCI [43]. SRT and Mini-Mental State Exam (MMSE) instruments were administered as part of this referring study, but not as part of the AG026158. Furthermore, Aβ levels were not obtained in the AG041795 study and thus were not available for participants drawn from that study.

Demographics are shown in Table 1.

**Table 1:** Demographics (mean ± sd)

Group	Amyloid-negative (*n* = 41)	Amyloid-positive (*n* = 16)	Mild cognitive impairment (*n* = 15)
**Age (years)**	66.7 ± 3.4	66.6 ± 4.4	65.9 ± 5.1
**Gender (F/M)**	19/22	6/10	6/9
**SRT total score**	49.7 ± 8.4	49.1 ± 10.8	39.7 ± 13.2*
**Mattis Dementia Rating Scale**	140.5 ± 2.2	138.8 ± 2.2*	–
**Mini-Mental State Exam**	–	–	27.6 ± 1.7

^*^p < .05 vs amyloid-neg

### Visual event-related potentials (vERPs)

**Fig. 1:**
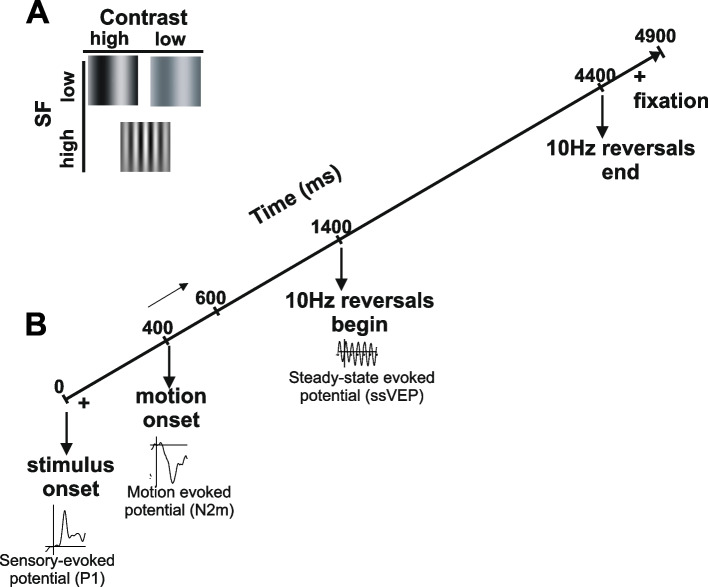
Schematic diagram of the optimized visual stimulation paradigm. **A** Stimuli used. **B** Timing of stimulus presentation

Participants were assessed using the recently developed optimized “JH-Flicker” visual paradigm [25, 28] that uses interleaved stimulus-onset, motion-onset, and ssVEP to evaluate the multiple aspects of early visual processing in parallel (Fig. 1). Each trial began with the onset of one of three types of patterns (vertical gratings): (1) LSF (0.8 cycles per degree (cpd)) at high (75%) luminance contrast (LSF_HC_), (2) LSF at low (8%) contrast (LSF), or (3) HSF (5 cpd) at high (75%) contrast (HSF_HC_). Motion onset began 400 ms after stimulus onset. The grating drifted rightward for 200 ms and remained static for 800 ms. Following, the stimulus counterphase reversed (10 Hz) for 3000 ms yielding a ssVEP response. Throughout the trial, participants maintained fixation on a central crosshair and pressed a button using their right index finger when it dimmed slightly.

The ongoing EEG was recorded using a 64-channel actiCAP cap (Easycap GmbH, Herrsching, Germany) with standard 10/10 system electrode placement [44] connected to a Brain Vision (BrainAmp) amplifier (Brain Vision LLC, Morrisville NC 27,560). Impedances were maintained below 10 kΩ. Data were digitized at 500 Hz, filtered offline (0.1–80 Hz), epoched from − 1000 to 3000 ms surrounding the onset and motion onset of each stimulus prior to averaging across trials and re-referenced to the average of all electrodes. Epochs with amplitudes exceeding ± 100 µV at any electrode, or those preceded/followed by a response, were excluded. A total of 420 trials (140 per stimulus type) were presented, corresponding to a 34.3-min total recording time. On average, 7.2% (Aβ −), 12.8% (Aβ +), and 9.1% (MCI) trials were excluded. Averaged vERPs were analyzed separately for each stimulus type. All analyses were performed using MATLAB (Mathworks, Natick, MA) with the EEGLAB and ERPLAB toolboxes [45].

Time–frequency (evoked-power) measures were obtained by convolving the time-domain averaged vERPs with a 3-cycle Morlet wavelet over a 3-s window beginning 1 s before onset, as described previously [28]. Evoked power was extracted at each time point over 74 frequency scales from 0.48 to 27.6 Hz, incremented logarithmically. Statistical analyses were carried out in the theta (4–7 Hz) and delta (1–4 Hz) frequency bands for stimulus-onset and motion-onset activity, respectively. Measurement latency windows were centered around the peak amplitude based on combined data from all participants, yielding a theta measurement window of 150–250 ms post-stimulus onset and a delta window of 50–250 ms post-motion onset.

ssVEPs were analyzed from 0 to 3000 ms following the onset of the first reversal. The total power spectrum of the ssVEP waveform was derived from the single-trial epochs via fast-Fourier transform (FFT) using a Hamming filter and averaged separately for each stimulus type. The mean FFT power at 10 Hz was computed for each electrode/stimulus. For each subject, values were averaged across the three stimulus types for each of the measures (theta, delta, ssVEP), yielding a single theta, delta, and ssVEP value per subject. We did not explore vERP power in other frequency bands (alpha, beta, gamma) because of a lack of activity in these bands both in our prior [25, 28] and present studies.

### Amyloid scans

Amyloid (Aβ) levels in the brain may be detected by positron emission tomography (PET) using a ligand such as florabetaben that binds to amyloid plaques and that can be labeled with a positron-emitting isotope (^18^F). PET scans can thus quantify both the degree of binding and the location of ligand binding within the brain following injection [46].

For this study, ^18^F-florbetaben was donated by Piramal (Piramal Pharma Inc.). PET scans were performed using a Siemens MCT PET/CT scanner in dynamic, three-dimensional acquisition mode. Dynamic acquisition frames were obtained over 20 min (4 × 5 min frames) beginning 50 min following the bolus injection of 10 mCi of ^18^F-florbetaben. An accompanying structural CT scan (in-plane resolution = 0.58 × 0.58 mm, slice thickness = 3 mm, FOV = 300 × 300 mm, number of slice = 75) was acquired and used for attenuation correction. PET data were reconstructed using a TrueX (HD-PET) algorithm. Images were smoothed with a 2-mm Gaussian kernel with scatter correction.

Dynamic PET frames (4 scans) were aligned to the first frame using rigid-body registration, and a static PET image was obtained by averaging the four registered frames. The static PET and CT images were coregistered and merged to generate a composite image in the PET static space. Each individual’s structural T1 image in FreeSurfer space was also registered to the participant’s merged image to transfer ROIs (see below) and the cerebellar gray matter from FreeSurfer space to static PET image space. These ROIs in static PET space were used to extract the regional PET data.

The standardized uptake value ratio (SUVR) was calculated as the ratio of amyloid deposition in the cortex versus the cerebellum, with values greater than 1 reflecting higher cerebro-cortical vs. cerebellar amyloid deposition. Individuals were characterized as Aβ + vs. Aβ − based on a ratio of > 1.25 [47]. Absolute SUVR levels were used for correlational analyses. The SUVR value was missing for one individual.

### The Awareness of Social Inference Task (TASIT) [29]

The TASIT assessment was added part-way through the study based on parallel findings in schizophrenia [35] and thus was available only for a subset of participants (*n* = 25). Procedures are as recently reported. Briefly, participants view a series of videos of social interactions. In each video, the main actor either makes a statement that is verifiably false based on events on the screen or sarcastic as reflected in facial expression and tone of voice. Viewers make a forced choice of lie vs. sarcasm after each trial. In other neuropsychiatric disorders, deficits in early visual processing contribute to impairments in social cognition [25, 36, 48], leading us to investigate a potential similar relationship with Aβ deposition.

### Statistics

One-way ANOVA was carried out on theta- and delta-evoked power with group (Aβ + , Aβ − , and MCI) as a between-participant factor with the post hoc Sidak tests, which controls for family-wise error rate across the multiple groups. Theta- and delta-evoked power were arc-tan transformed prior to analysis to improve normalization. ssVEP data were log-transformed. Effect sizes were calculated and interpreted according to the conventions of Cohen [49]. A composite measure was defined by linear regression of the separate theta, delta, and ssVEP measures vs. SUVR in the healthy participant group. Prediction analyses utilized the calculated composite measure. A leave-one-out cross-validation (LOOCV) was conducted in order to assess model stability. All statistics were two-tailed with preset *α* level for a significance of *p* < 0.05.

## Results

**Fig. 2:**
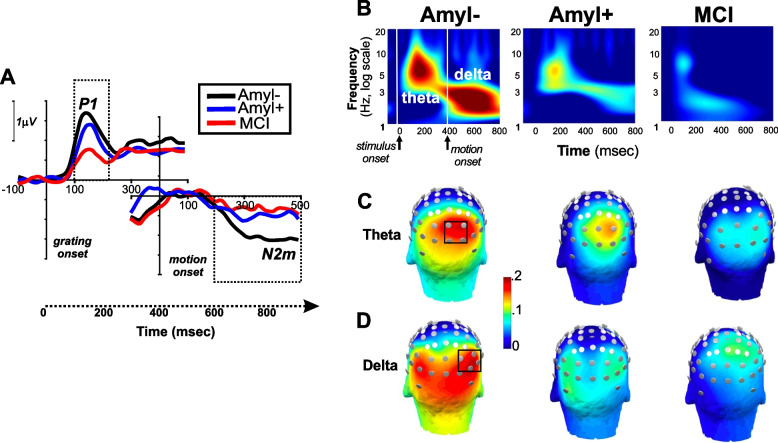
Neurophysiological results. **A** vERP elicited by stimulus grating onset (P1) and motion onset (N2m) onset relative to respective pre-stimulus baseline. **B** Time–frequency (ERP) response to stimulus onset (theta, 4–7 Hz) and motion onset (delta, 1–4 Hz). **C** Head map of stimulus-onset theta activity by group. **D** Head map of motion-onset delta activity by group

Participants included 57 cognitively intact participants(41 Aβ − /16 Aβ +) and 15 participants with documented MCI. Responses were obtained to both stimulus- and motion-onset activity and analyzed using both time- (Fig. 2A) and TF-domain (Fig. 2B) analyses. As expected, primary activity occurred within the theta (Fig. 2C) and delta (Fig. 2D) frequency bands and was maximal over occipital visual regions in all groups.

**Fig. 3:**
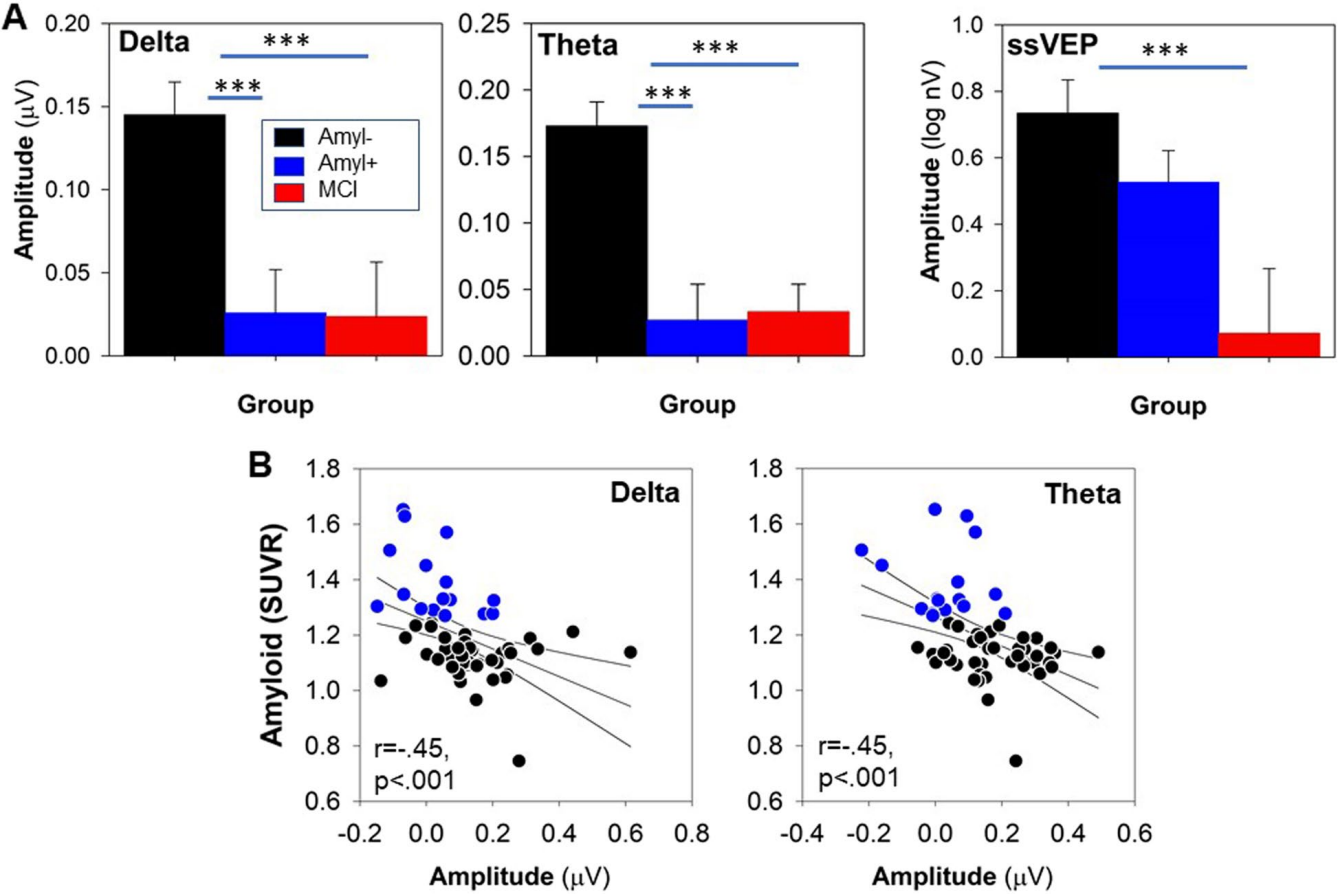
Between-group comparisons of indicated vERP components. **A** Theta response to stimulus onset. **B** Delta response to motion onset. **C** ssVEP response. **D** Correlation between theta and amyloid level. **E** Correlation between delta and amyloid levels. **p < .01; ***p < .001

Also, as predicted, theta (*F*_2,69_ = 15.3, *p* < 0.001), delta (*F*_2,69_ = 8.60, *p* < 0.001), and ssVEP (*F*_2,69_ = 6.30, *p* = 0.003) amplitudes were all significantly different across the groups (Fig. 3A). For both theta and delta measures, post hoc testing showed highly significant differences between Aβ − individuals with intact neurocognitive performance and both of the other groups (all *p* < 0.005).

Effect sizes for differentiation of Aβ − vs. Aβ + participants were large for both theta (*d* = 1.17, *p* < 0.001) and delta (*d* = 0.91, *p* = 0.001) measures. Effect sizes were also large for theta (*d* = 1.16, *p* < 0.001) and delta (*d* = 0.87, *p* = 0.002) measures between neurocognitively intact Aβ − and MCI participants. For ssVEP, Aβ − and Aβ + healthy elders did not differ significantly (*d* = 0.33, *p* = 0.22). However, MCI patients showed significant reductions vs. neurocognitively intact Aβ − individuals (*d* = 0.89, *p* = 0.002). Across participants, significant correlations were observed between both theta and delta responses and amyloid levels (Fig. 3B).

### Categorical analyses

**Fig. 4:**
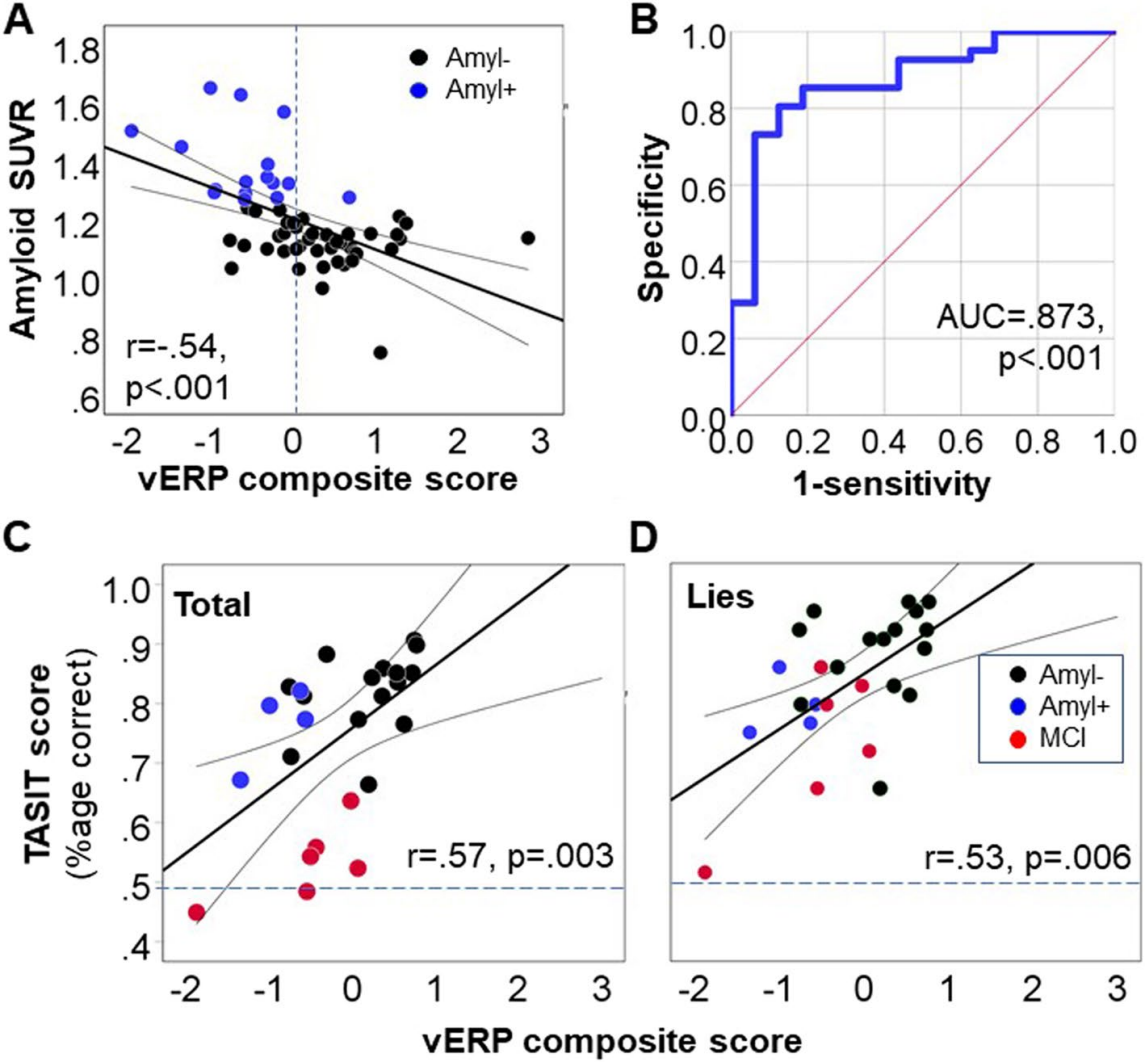
Predictive utility. **A** Correlation between composite vERP measure and amyloid values. Composite measure was calculated as 3.70 × theta + 3.41 × delta + .02 × ssVEP − .7). The contributions of both theta (*p* = .021) and delta (*p* = .028) were independently significant. **B** ROC curve for separation of amyloid-negative vs. positive participants based on composite vERP measures. **C**, **D** TASIT performance for amyloid-positive and negative healthy elders to sarcasm total scores (**C**) and lie (**D**) trials

In order to determine the degree to which vERP could be used to differentiate Aβ − from Aβ + participants, we calculated a composite score based on simultaneous regression of theta, delta, and ssVEP values vs. amyloid SUVR (Fig. 4A). ROC analyses for differentiation of Aβ − from Aβ + healthy elders (Fig. 4B) showed highly significant discrimination effects, such that a cutoff of < 0 on the composite scale correctly identified 15 of 16 (93.8%) of amyloid-positive participants and 13 of 15 (86.7%) MCI participants, while rejecting 30 of 41 (73.2%) amyloid-negative participants (LR*χ*^2^ = 32.4, df = 2, *p* < 0.0001).

The overall correct classification of amyloid-positive vs. amyloid-negative participants was 78.9% (*χ*^2^ = 19.2, *p* < 0.001). Although it was not possible to use separate training and testing sets given the sample size, the accuracy remained unchanged (78.9%) in the LOOCV analysis.

### Relationship with TASIT

TASIT was added part-way through the project based on emergent findings from our parallel schizophrenia studies [35], so that TASIT data were available from only 19 neurocognitively intact (15 Aβ − , 4 Aβ +) and 6 MCI participants. Across the groups, TASIT total (*r* = 0.53, *p* = 0.006) and lie (*r* = 0.58, *p* = 0.002) scores correlated significantly with the composite index (Fig. 4C, D). Among the component scores, the strongest correlations were observed for the stimulus-onset theta response relative to both TASIT total (*r* = 0.60, *p* = 0.001) and lie (*r* = 0.54, *p* = 0.006) scores. Significant independent correlations were also observed with ssVEP (total: *r* = 47, *p* = 0.017; lie: *r* = 0.44, *p* = 0.04). Although TASIT scores differed significantly across the groups (*F*_2,22_ = 40.3, *p* < 0.001) with MCI participants showing worse performance than other groups (*p* < 0.001), the correlations between vERP and TASIT total score remained significant following control for group status (*F*_1,21_ = 6.44, *p* = 0.019).

### Control analyses

Neurocognitive test measures for the groups are shown in Table 1. As expected, there was an overall difference in cognitive performance as measured by the SRT across groups (*F*_2,63_ = 4.00, *p* = 0.023), with MCI participants showing significant impairments relative to Aβ − participants (Sidak *p* = 0.02) but no significant difference vs. Aβ + (Sidak *p* = 0.99). In contrast, Aβ − and Aβ + participants showed a significant difference in DRS (*F*_1,55_ = 7.11, *p* = 0.01).

Correlations between vERP and cognition scores were all non-significant (*p* > 0.2). Furthermore, between-group differences in vERP measures remained strongly significant even following control for SRT (*F*_2,62_ = 14.8, *p* < 0.0001) or DRS (*F*_1,54_ = 30.8, *p* < 0.0001). Correlations between cognition scores and amyloid levels were also non-significant (*p* > 0.3). Moreover, the correlations between vERP and Aβ SUVR remained significant following covariation for neuropsychological performance (*r*_*p*_ =  − 0.53, df = 54, *p* < 0.0001).

There were no significant differences in age or gender composition across the groups (Table 1). No differences were observed by age or gender on any of the experimental measures.

## Discussion

Abnormalities in RGC function have been increasingly documented in early AD and shown to differentiate Aβ + from Aβ − older individuals [14, 15]. To our knowledge, this is the first study to investigate cortical vERP in a similar population. Consistent with recent ERG results, presymptomatic AD subjects were distinguishable from amyloid-negative individuals with an accuracy of ~ 80%.

Deficits were observed to both stimulus onset and motion, suggesting potential involvement of the visual magnocellular system. Similar deficits were observed in a group of MCI individuals. Furthermore, the deficits correlated with impaired social cognition, as reflected in performance on the TASIT, which has previously been linked to increased social isolation and loneliness even in otherwise cognitively intact individuals. These findings thus support and amplify the relevance of early visual dysfunction for both detection and functional assessment of older individuals at potential risk for AD.

### vERP in AD

Although vERPs have previously been studied in individuals with established MCI or AD (e.g., [50–52]), to our knowledge, this is the first study to demonstrate vERP deficits associated with amyloid levels in pre-symptomatic individuals. To the extent that prior studies have been conducted, deficits have been observed primarily in later stages of visual processing, such as the higher-level processing within the dorsal visual stream, whereas earlier stages of processing were intact (rev. in [53, 54]).

For example, one set of studies used wide-field changes of luminance from 10 to 40 cd/m^2^ during a visual oddball paradigm and found deficits in both theta and delta power already in the MCI state, whereas evoked delta amplitude showed a deficit only in AD and correlated with neuropsychological test performance [16, 55]. Future studies are needed to address the relative information obtained from the different stimulation procedures.

Our findings also encourage further study of motion-induced responses within both preclinical- and clinical-stage AD. Prior studies using optic flow stimuli have shown vERP deficits in higher-tier dorsal regions that correlated with neuropsychological impairments [56–61]. Deficits have been observed as well in motion-related fMRI activation of the primary visual cortex [62]. Interestingly, vERP abnormalities beginning in the retina have also been observed in 5xFAD transgenic [63] and other [64] mice in association with amyloid deposition and in zebrafish cholinergic models [65], suggesting potential translational utility.

Of note, our findings of large effect size differences in vERP measures (*d* = 1.2–1.8) also contrast sharply with the much smaller effect size differences observed with neuropsychological performance assessed with such measures as the Mattis Rating Scale or the Bushke Selective Reminding Task in our participants (*d* = 0.33). Other groups have also found relatively modest neurocognitive deficits in early preclinical AD. For example, in one recent meta-analysis of 57 studies of neurocognition in amyloid-positive healthy elders, effect sizes were in the range of 0.12 to 0.20 [66]. The lack of association with cognitive measures suggests that vERP may have utility in preclinical cases where the cognitive assessment may not by itself be very useful in predicting the likelihood of AD.

Other forms of neural synchrony related to visual system dysfunction may also be altered in AD. For example, hyposynchronous alpha oscillations over the visual cortex have been found to colocalize with TAU deposition and correlate with the degree of global cognitive dysfunction, while hypersynchronous delta-theta oscillations over frontal and anterior temporoparietal cortices colocalized with both TAU and Aβ [67]. Future studies should address the degree to which abnormalities in resting brain oscillations correlate with vERP impairments.

### Functional consequences of early visual dysfunction

In addition to showing the potential utility of time–frequency vERP as a surrogate biomarker for amyloid burden, the present study raises the possibility that deficits in early visual processing may be an overlooked cause of social disability in both Aβ + and MCI individuals. In schizophrenia, deficits in early visual processing contribute significantly to impaired face processing and social cognition, as shown by tests such as the Penn face emotion recognition test and TASIT [34–36, 68–70]. Similarly, visual deficits are associated with diverse aspects of cognitive dysfunction in MS [24].

Although visual sensory tests have not been extensively studied in healthy aging, several studies have demonstrated a decline in social cognition using tests such as TASIT that were independent of more general cognitive impairments [37–40] as in the present study. Future studies incorporating both visual and social cognitive measures are needed to evaluate the potential contributions of early visual dysfunction to social cognitive impairments in healthy aging, similar to what is observed in schizophrenia.

### Limitations

Despite the robust deficits in vERP observed in this study, several limitations must be acknowledged. First, the sample size was small although commensurate with that of recent ERG studies (e.g., 14, 15), and no longitudinal follow-up was incorporated. Thus, it remains to be determined whether correlations will be observed longitudinally as well as replicated cross-sectionally. We also did not assess the cortical TAU levels, which might modulate the effects of amyloid on neurocognition [66]. Several aspects of our methods, such as artifact rejection, pre-stimulus intervals, post hoc normalization procedures, and calculation of a composite score were used to optimize the dataset and likely affected the specific pattern of the result. Replication in an independent cohort with pre-specified measures is therefore critical.

Amyloid imaging was not done in the MCI group, leaving open the degree to which the observed deficits in that group are related to amyloid or to other pathophysiological processes. Rates of Aβ positivity among MCI individuals aged 60–70 are estimated at ~ 50% [71]. In our study, MCI participants showed a somewhat different profile from Aβ + individuals without memory impairments, in that they showed deficits in ssVEP generation that were not present in the non-MCI groups. These findings highlight the utility of the interleaved approach for distinguishing between the groups and emphasize the need to expand the present sample to include a wider range of individuals (e.g., normal cognition, subjective cognitive decline, MCI, AD) with full biomarker assessment. Indices developed for between-group separation were derived from the same population. Thus, although they were validated using leave-one-out cross-validation, they need to be independently replicated.

Finally, the correlations with TASIT were performed only on a subgroup of individuals across diagnostic groups and must be considered exploratory. Furthermore, whereas deficits in social cognition are linked to loneliness and isolation within the aging literature, no specific measures related to these constructs were collected in the present study. Thus, replication of these findings in an independent data sample with expanded behavioral measures is critical.

## Conclusions

Although Aβ deposition is known to contribute to degeneration in the preclinical phase of AD, there are at present few physiological measues sensitive to the deposition. Here, we show that optimized time–frequency-based vERP approaches are sensitive to amyloid deposition even in otherwise healthy individuals and that amyloid effects on early visual processing may contribute to previously documented impairments in social cognition. vERP measures are easy to implement, have high test–retest reliability, and can be followed longitudinally if needed. Time–frequency analyses, while more complex than traditional time-domain approaches, nevertheless have become increasingly tractable for routine analytic use. Further studies to evaluate the utility of these biomarkers in early detection, intervention, and longitudinal follow-up studies are warranted. In addition, parallel studies of visual processing and social cognition are needed, to evaluate the potential contribution of visual processing impairments to social functional deficits in aging.

## Data Availability

The raw datasets used and/or analyzed during the current study are available from the corresponding author upon reasonable request.

## Abbreviations

Aβ: Amyloid-beta
AD: Alzheimer’s disease
AUC: Area under the curve
EEG: Electroencephalogram
ERG: Electroretinogram
GCL: Ganglion cell layer
MCI: Mild cognitive impairment
OCT: Optical Coherence Tomography
SRT: Selective reminding task
ssVEP: Steady-state visual-evoked potential
TASIT: The Awareness of Social Inference Test
TF: Time–frequency
vERP: Visual event-related potential
